# Supplementary material for: Diagnosis and Management of Type 1 Sialidosis: Clinical Insights from Long-Term Care of Four Unrelated Patients
Source: Brain Sci. 2020 Aug 1;10(8):506. doi: 10.3390/brainsci10080506 (PMC7465165; doi:10.3390/brainsci10080506)
Supplement: Supplementary file 1 [file brainsci-10-00506-s001.pdf]

Supplementary Materials:

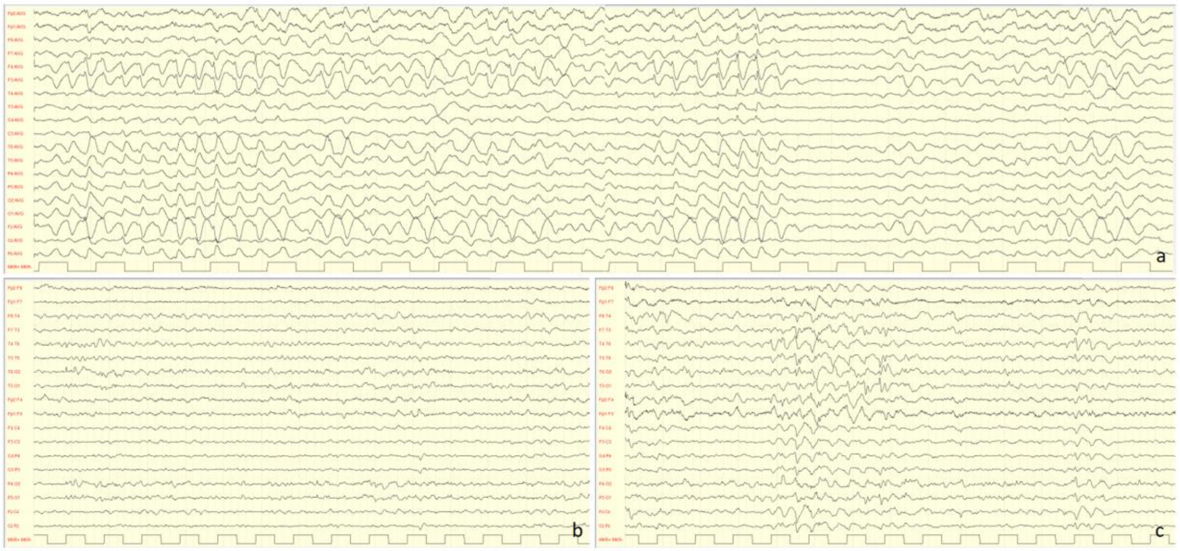

Figure S1. Electroencephalography of patient #1. (a) EEG performed at the age of 22, showing high amplitude and diffuse spike and slow-wave discharges that were more evident over the frontal areas. (b) EEG of patient #1 performed at the age of 42 years old, showing a dominant theta (6-7 Hz) background activity and (c) high voltage delta discharges intermixed with epileptiform spikes and polyspike-wave discharges over the temporal areas.

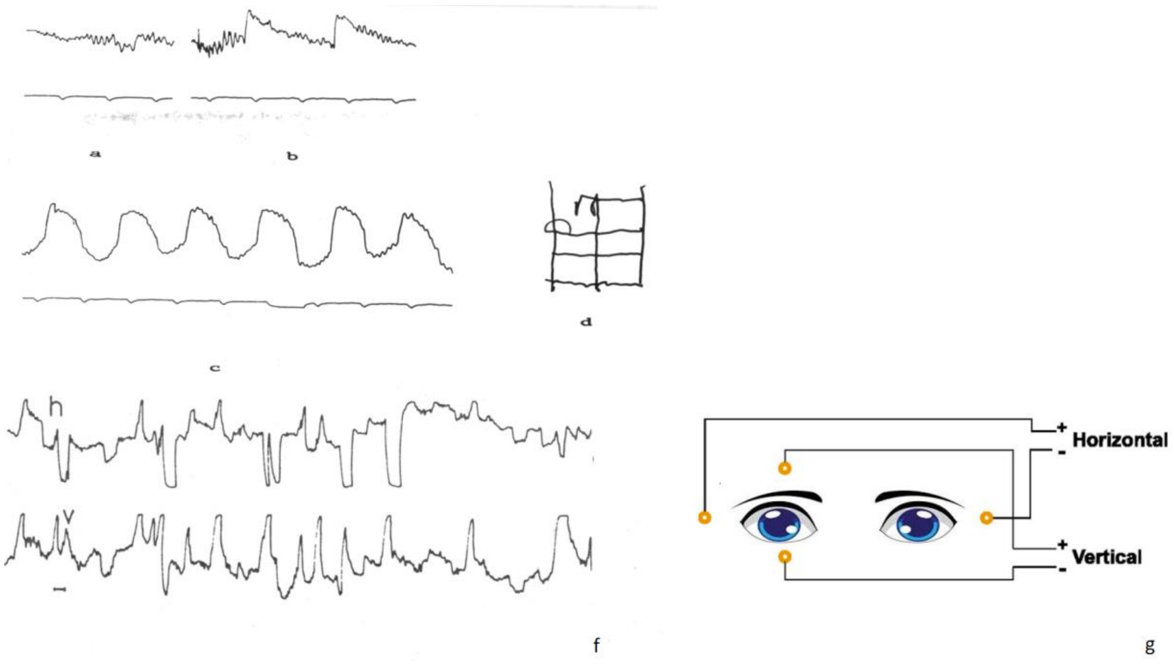

Figure S2. Electrooculography of patient #1. Electrooculography performed at the age of 15, showing a broad flutter in primary eye position consistent with oscillopsia (a); hypermetric saccades intermixed with flutter, consistent with pendular nystagmus (b); slow eye movement with superimposed flutter (c). (d) The image shows tremor at the graphic task at the same age. (f) Electrooculography (top trace h: horizontal; bottom trace v: vertical; electrodes are placed as shown in image (f)) performed at the age of 21 and showing continuous high amplitude multidirectional saccades (eye dancing) consistent with opsoclonus.
